# Palmitic acid promotes resistin-induced insulin resistance and inflammation in SH-SY5Y human neuroblastoma

**DOI:** 10.1038/s41598-021-85018-7

**Published:** 2021-03-08

**Authors:** Hamza Amine, Yacir Benomar, Mohammed Taouis

**Affiliations:** 1 Molecular Neuroendocrinology of Food Intake (NMPA), CNRS UMR 9197, University of Paris-Saclay, Orsay, France; 2 NMPA, Department of Development, Evolution and Cell Signaling, CNRS UMR 9197, Paris-Saclay Institute of Neurosciences (NeuroPSI), Orsay, France; 3 Functional Genomics and Cancer, IGBMC, Illkirch, France

**Keywords:** Cell signalling, Cellular neuroscience, Molecular neuroscience

## Abstract

Saturated fatty acids such as palmitic acid promote inflammation and insulin resistance in peripheral tissues, contrasting with the protective action of polyunsaturated fatty acids such docosahexaenoic acid. Palmitic acid effects have been in part attributed to its potential action through Toll-like receptor 4. Beside, resistin, an adipokine, also promotes inflammation and insulin resistance via TLR4. In the brain, palmitic acid and resistin trigger neuroinflammation and insulin resistance, but their link at the neuronal level is unknown. Using human SH-SY5Yneuroblastoma cell line we show that palmitic acid treatment impaired insulin-dependent Akt and Erk phosphorylation whereas DHA preserved insulin action. Palmitic acid up-regulated TLR4 as well as pro-inflammatory cytokines IL6 and TNFα contrasting with DHA effect. Similarly to palmitic acid, resistin treatment induced the up-regulation of IL6 and TNFα as well as NFκB activation. Importantly, palmitic acid potentiated the resistin-dependent NFkB activation whereas DHA abolished it. The recruitment of TLR4 to membrane lipid rafts was increased by palmitic acid treatment; this is concomitant with the augmentation of resistin-induced TLR4/MYD88/TIRAP complex formation mandatory for TLR4 signaling. In conclusion, palmitic acid increased TLR4 expression promoting resistin signaling through TLR4 up-regulation and its recruitment to membrane lipid rafts.

## Introduction

Inflammation is a hallmark of several metabolic diseases including obesity and type 2 Diabetes (T2D) as evidenced for instance by increased levels of pro-inflammatory cytokines in subjects with T2D^1^. Furthermore, the deleterious effects of pro-inflammatory factors on insulin responsiveness and signaling have been largely described in different tissues and cell models including hepatocytes, muscle cells and adipocytes^2–4^. Beside, obesity is considered as a risk factor for T2D and both diseases are known to promote low-grade inflammation state. Additionally, among other factors involved in obesity/T2D relationship, the increased circulating levels of free fatty acids (FFAs) in obese subjects. Indeed, obese and T2D subjects exhibit a high plasma levels of non-esterified FFAs especially palmitic acid, the most abundant saturated fatty acid in the blood. Palmitic acid has been described as a negative modulator of insulin signaling through the inhibition of insulin action in isolated hepatocytes, myocytes and adipocytes^5–7^. Furthermore, it has been also reported that the exposure to palmitic acid induced a lipotoxicity of pancreatic β cells leading to the alteration of pancreatic β cell functions. Emerging evidence suggests that the alteration of whole body insulin responsiveness is initiated in the brain and precisely in the hypothalamus^8,9^. HFD promotes hypothalamic resistance towards insulin and leptin, main anorexigenic hormones, through an inflammatory-dependent mechanism disturbing then energy homeostasis control^10^. Accumulated evidences suggested that FFAs might exert their lipotoxic effects through Toll-like receptors (TLRs) especially TLR4^11^. Indeed, FFA-dependent inflammation responses are mediated by TLR4 signaling cascade, suggesting that saturated fatty acids may directly or indirectly bind to TLR4^12^. Furthermore, FFAs are able to activate TLR4 signaling in monocytes and adipocytes^13^. Among other ligands of TLR4, LPS is the most known to promote inflammation through this signaling pathway^14^. We have reported that resistin was able to activate neuroinflammation and to promote insulin resistance through its binding to TLR4^15^. Resistin is a key metabolic regulator in obesity and insulin resistance. Indeed, resistin has been identified as proinflammatory and insulin resistance promoting factor. It is expressed in mice white adipose tissue (WAT), whereas in human its expression in WAT is weak but high in immune cells (monocytes and macrophages)^16,17^. Thus, resistin could be the metabolic link between obesity and insulin resistance, since resistin levels are high in inflammatory states such as in obese subjects promoting then insulin resistance. Furthermore, it has been also shown that palmitic acid increased the release of resistin from differentiated 3T3-L1 adipocytes^13^. We have shown that central resistin impaired insulin signaling and increased pro-inflammatory cytokines’ expression at both hypothalamic and peripheral levels^15^. These effects were mediated through newly identified resistin/TLR4 signaling pathway^15^. According to these data, we hypothesize that palmitic acid or saturated FFAs effects regarding inflammation and insulin resistance could be, at least partially, indirect through the augmentation of resistin plasma levels promoting then neuro-inflammation and overall inflammation. Indeed, palmitic acid and saturated FFAs have been described as endogenous ligand for TLR4 but however, so far there is no evidence for a direct binding of FFAs to TLR4. Here, we investigated the impact of palmitic acid on insulin responsiveness and its potential effects on TLR4 expression and resistin action in human SH-SY5Y neuroblastoma cell line.

## Materials and methods

### Cell culture and stimulation

SH-SY5Y human neuroblastoma cells were grown in Dulbecco’s modified Eagle’s medium (DMEM) supplemented with 10% fetal calf serum (FCS), 2 mmol L-glutamine, 100 U/mL penicillin and 100 µg/mL streptomycin in a 37 °C, 5% CO2 humidified atmosphere. Serum starved cells were then incubated for 4 h in the presence or absence of palmitic acid (200 µM), DHA (20 µM), resistin (200 ng/mL) or insulin (100 nM) and/or challenged for 15 min with insulin (100 nM), to evaluate the impact of fatty acids and resistin treatment on SH-SY5Y insulin responsiveness, or with resistin (200 ng/mL), to analyze the effect of fatty acid treatment on resistin/TLR4 signaling. MK-2206 (Akt inhibitor), U0126 (MEK1/2 inhibitor), SP600125 (JNK inhibitor), SB202190 (p38 MAPK inhibitor) were provided by Santa Cruz Biotechnology and were dissolved in DMSO for cell culture experiments, the control cells were treated with DMSO alone. Cells were exposed to 1 µM MK-2206, 10 µM U0126, 20 µM SP600125, 50 µM SB202190 for 1 h and then after washout cells were treated with resistin (200 ng/mL) for 4 h.

### Fatty acids preparation

27.8 mg sodium palmitic acid was dissolved in 1 mL sterile water to make 100 mM palmitic acid stock solution. DHA was suspended in absolute ethanol to make 10 mM DHA stock solution. 10% free fatty acid low endotoxin bovine serum albumin (FFA-BSA) was prepared in sterile PBS. Palmitic acid and DHA were conjugated to FFA-BSA in a 6:1 molar ratio. The solutions were then filtered through a 0.2 µm filter and stored at -20 °C. FFA-BSA/fatty acids complexes were further diluted in serum free DMEM to reach the desired final concentrations and were shaken for 1 h at 140 rpm at 40 °C before use. FFA-BSA alone was used as a control.

### Western blot analysis

After treatment, SH-SY5Y cells were solubilized in lysis buffer containing 20 mM Tris–HCL (pH 7.5), 137 mM NaCl, 1 mM MgCl2, 1 mM CaCl2, 1% nonidet P40, 10% glycerol, protease inhibitors (0.35 mg/ml PMSF, 2 µg/ml leupeptin and 2 µg/ml aprotinin) and phosphatase inhibitors (10 mM sodium fluoride, 1 mM sodium orthovanadate, 20 mM sodium β-glycerophosphate and 10 mM benzamidine). Lysates protein concentration was determined using the BCA Protein Assay Kit (Thermo Scientific, Illkirch, France) and analyzed by Western blot. Briefly, proteins were subjected to SDS-PAGE and transferred onto Immobilon-FL membrane (Life Technologies, Illkirch, France). Blots were then blocked with 5% Bovine Serum Albumin fraction V (BSA) (Euromedex, souffelweyersheim, France) and incubated with primary antibodies raised against p-AKT, AKT, p-P38MAPK, P38MAPK, pERK1/2, ERK1/2, Insulin receptor (IR) β subunit, β-tubulin (Cell signaling, Danvers, MA, USA), Toll-like receptor 4 (TLR4), MyD88, TIRAP (Santa Cruz Biotechnology, Dallas, TX, USA) and resistin (Abcam, Cambridge, UK) overnight at 4 °C. The immunoblots were washed with PBS, incubated with the appropriate secondary antibody labeled with Alexa 488 (Life Technologies, Illkirch, France) then scanned and quantified using the Brucker Molecular Imaging System 4000MM Pro (Billerica, MA, USA).

### Immunoprecipitation

Protein lysates were incubated with antibodies raised against TLR4 overnight at 4 °C. The immune complexes were precipitated after incubation with a protein A-agarose (Sigma-Aldrich, St. Quentin Fallavier, France) for 2 h at 4 °C and then heated in loading buffer at 100 °C for 5 min. Western blots were performed as described before and membranes were immunoblotted with antibodies raised against TLR4, MyD88 or TIRAP.

### RNA extraction and quantitative real-time RT-PCR

SH-SH5Y cells were solubilized in TRIzol LS reagent (Invitrogen, Illkirch, France) and total RNA was extracted according to the manufacturer’s recommendations. 1 µg of total RNA was reverse transcripted (F-572L M-Mulv) (Finnzymes, Illkirch, France), and the cDNAs were subjected to real-time PCR using the Step One apparatus (Applied Biosystems, Illkirch, France), Fast SYBR Green Master Mix (Applied Biosystems, Illkirch, France) and adequate primers: GAPDH forward, 5′-ACTCCGACCTTCACCTTCCCC-3′, and reverse, 5′-CTCCCGCTTCGCTCTCTGCT-3′; IL6 forward, 5′-TGCTCCTGGTGTTGCCTGCT-3′, and reverse, 5′-TGAGTGGCTGTCTGTCTG-GGG-3′; InsR forward, 5′-GAGAAGGTGGTGAACAAGGAGTC-3′, and reverse, 5′-CCGTGAA-GTGTCGCAAGCC-3′; TLR4 forward, 5′-GACTTGCGGGTTCTACATCA-3′, and reverse, 5′-CA-TAGGGTTCAGGGACAGGT-3′; TNFα forward, 5′-GCTCCAGGCGGTGCCTTGTTC-3′, and reverse, 5′-AGGCTTGTCACTCGGGGTTC-3’.

### Small interfering RNA silencing

Negative-control scrambled Silencer Select small interfering RNA (siRNA), TLR4-targeting Silencer Select siRNA, and MyD88 targeting Silencer Select siRNA were purchased from Ambion (Illkirch, France) and used following the manufacturer’s instructions. Briefly, SH-SH5Y cells were seeded at 1 $\times$ 10^6^ cells per well in a six-well plate and transfected with the different Silencer Select siRNA to a final concentration of 5 nM using the Lipofectamine 2000 (Invitrogen, Illkirch, France) transfection reagent according to the manufacturer’s instructions. 48 h after transfection, serum starved cells were treated for 4 h with palmitic acid (200 µM) or FFA-BSA as a control then challenged with resistin (200 ng/mL) for 15 min. Cells were then solubilized and protein lysates were subjected to Western blot as described before.

### Crosslinking experiments

Cross-Linking experiments were performed as previously described^12^. Briefly, serum starved SH-SH5Y cells were washed with ice-cold PBS then treated with resistin (200 ng/mL) for 1 h at 4 °C. BS3 crosslinking agent (Thermo Scientific, Illkirch, France) was then added directly to the incubation solution to a final concentration of 2.5 mM and followed by an additional incubation for 1 h at 4 °C. Crosslinking reaction was stopped by the addition of a quenching solution (20 mM Tris–HCL pH 7.5). Cells were solubilized and protein lysates were then subjected to immunoprecipitation using antibodies raised against TLR4. Western blots were performed as described before and membranes were immunoblotted with antibodies raised against TLR4 or resistin.

### Stable transfection and reporter gene luciferase assay

NF-κB luciferase reporter gene cells were generated by stably transfecting SH-SY5Y cells with the pGL4.32[*luc2P*/NF-κB-RE/Hygro] vector (Promega, Charbonnières les bains, France) containing five copies of an NF-κB response element that drives transcription of the luciferase reporter gene *luc2P* and a mammalian selectable marker for hygromycin resistance. After hygromycin selection (200 µg/mL), surviving cells were transfected with a control vector containing β-galactosidase with a cytomegalovirus promoter. 48 h after transfection, serum starved cells were treated 4 h with resistin (200 ng/ml), palmitic acid (200 µM) or DHA (20 µM) then luciferase and β-galactosidase activity were measured using the Mithras LB 940 apparatus (Berthold technologies, Bad Wildbad, Germany) and the Dual-Light Chemiluminescent Reporter Gene Assay System (Applied Biosystems, llkirch, France) according to the manufacturer’s instructions. Luciferase activity was normalized by β-galactosidase activity. All transfections were performed using Lipofectamine 2000 (Invitrogen, llkirch, France) according to the manufacturer’s instructions.

### Immunocytochemistry and microscopy analysis

SH-SY5Y cells were seeded onto Lab-Tek chamber Slides (Millipore, Darmstadt, Germany). Serum starved cells were treated 4 h with palmitic acid, DHA or FFA-BSA as a control, then washed with ice-cold PBS and incubated or not with 1 µg/mL of isothiocyanate-conjugated cholera toxin B subunit (FITC-CTxB) for 15 min at 4 °C. Cells were washed with PBS then fixed with 4% paraformaldehyde for 20 min at 4 °C followed by incubation with 50 mM NH_4_Cl for 20 min and were blocked/permeabilized with a solution containing 0.1% Triton X-100, 0.2% Fish Gelatin, 2% normal donkey serum for 1 h at room temperature. Fixed cells were then incubated with 1/300 dilution of rabbit anti-TLR4 (Santa Cruz Biotechnology, Dallas, TX, USA) in Blocking/permeabilization solution overnight at 4 °C. Cells were washed with ice-cold PBS and incubated with 1/500 dilution in PBS of Alexa Fluor 488 conjugated donkey anti-rabbit IgG (Life Technologies, Illkirch, France) or DyLight 649 conjugated donkey anti-rabbit IgG (Jackson ImmunoResearch, West Grove, PA, USA) for 1 h at room temperature. Cells were then washed with PBS and incubated for 3 min with 4,6-diamidino-2-phenylindole (DAPI) for DNA staining. After washing with PBS coverslips were mounted in Vectashield antifade mounting medium (Vectorlabs, Burlingame, Ca, USA) and slides were sealed with nail polish. Immunofluorescence was examined by confocal microscopy (Zeiss MRC1024ES; Zeiss microscopy, Jena, Germany) for TLR4 recruitment into lipid rafts.

### Hormones and chemicals

Human resistin was purchased from Shenondoah Biotechnology (Warwick, PA, USA). Human insulin Actrapid was provided by Novo Nordisk (Bagsvaerd, Denmark). Sodium palmitic acid, DHA and FFA-BSA were purchased from Sigma-Aldrich (St. Quentin Fallavier, France) and all cell culture reagents were purchased from Euromedex (Souffelweyersheim, France). All other chemicals were purchased from Sigma-Aldrich (St. Quentin Fallavier, France).

### Data analysis and statistics

Repeated-measures two-way ANOVA followed by Bonferroni *post-hoc* test were used to test the changes in signaling and gene expression experiments and *P* value $<$ 0.05 was considered as statistically significant. The results are expressed as means $\pm$ S.E.M.

## Results

### Palmitic acid reduced insulin-dependent Akt/ERK phosphorylation and increased TLR4 expression in SH-SY5Y neuroblastoma cells

**Figure 1:**
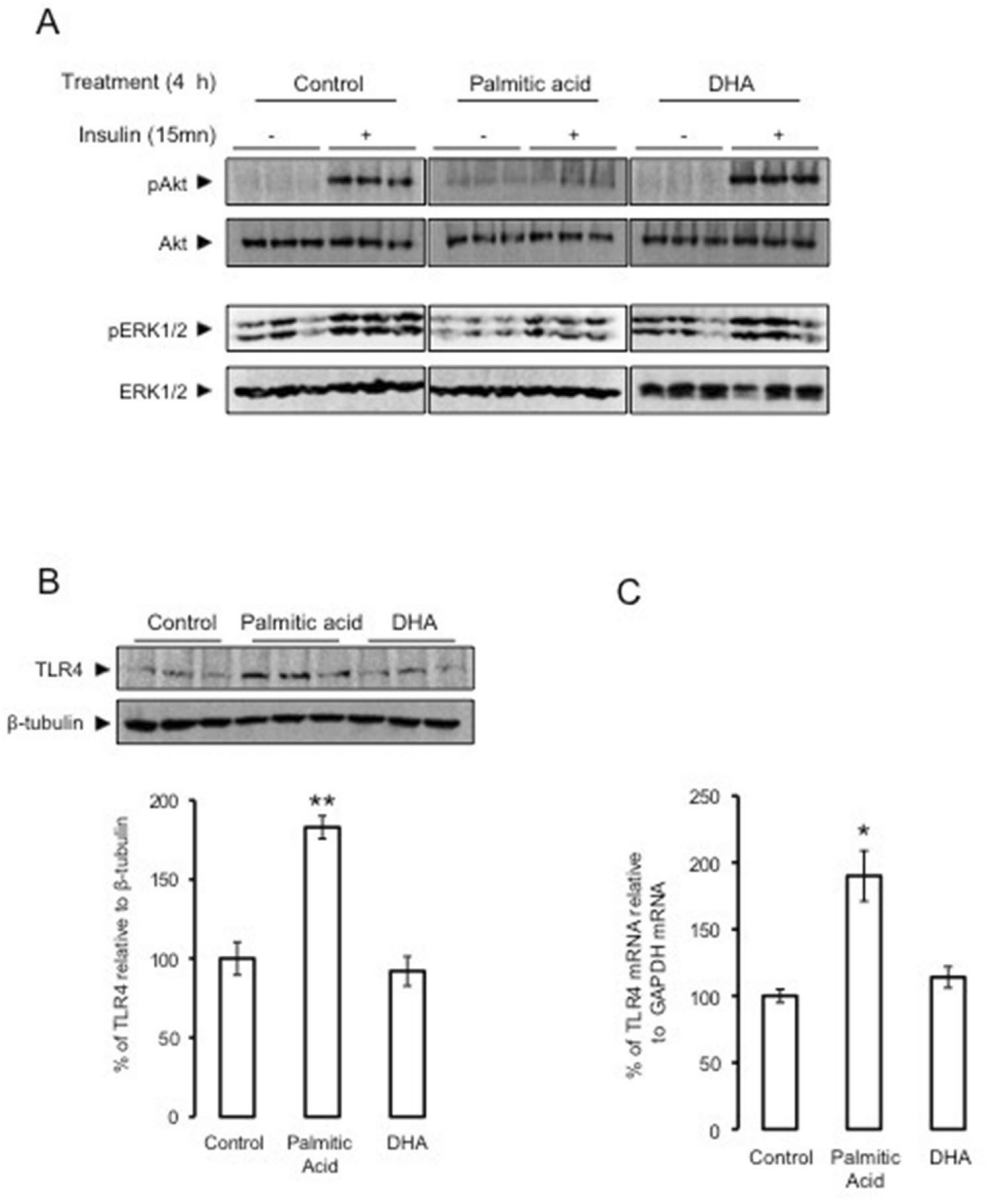
Palmitic acid but not DHA, inhibits insulin action and up-regulates TLR4. Human SH-Y5Y neuroblastoma cells were pretreated with palmitic acid, DHA or placebo during 4 h, then insulin-dependent Akt and ERK1/2 phosphorylation measured by Western blots using adequate antibodies, Control, Palmitic acid and DHA blots were performed in separate membranes, in each case the same membrane was blotted with different antobodies (panel **A**); TLR4 expression was measured by Western blot using anti-TLR4 antibodies and the expression was normalized to β tubilin, and band densities were quantified and expressed as ratio of TLR4/β-tubilin (panel **B**); TLR4 mRNA expression was determined by qRT-PCR normalized to GAPDH (panel **C**). Western blot and qRT-PCR data were presented as means ± SEM (n = 3), * and ** denoted significant differences vs control at *p* < 0.05 and *p* < 0.005, respectively.

To investigate the effect of palmitic acid on neuronal insulin responsiveness, SH-SY5Y cells were treated with palmitic acid, DHA or placebo. SH-SY5Y cells treated with palmitic acid exhibited lower insulin-dependent Akt and Erk1/2 phosphorylation as compared to untreated cells (Fig. 1A). In contrast, DHA treatment increased insulin-dependent phosphorylation of Akt and Erk1/2 (Fig. 1A). Since, we have reported that the activation of TLR4 signaling impaired insulin signaling, we investigated the effect of palmitic acid on TLR4 expression. Palmitic acid treatment increased the expression of TLR4 at both protein and mRNA levels in SH-SY5Y cells, whereas DHA treatment did not modify TLR4 expression (Fig. 1B,C).

### Palmitic acid increased the expression of pro-inflammatory markers in SH-SY5Y neuroblastoma cells

**Figure 2:**
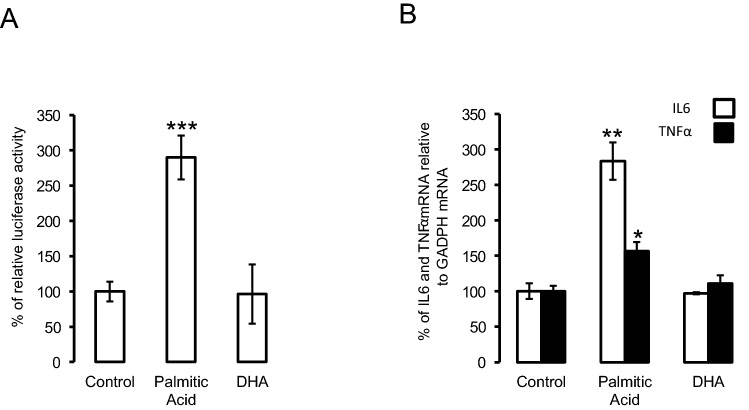
Palmitic acid but not DHA, increased the expression of pro-inflammatory factors. (Panel **A**) human SH-SY5Y neuroblastoma cells stably tranfected with NF-κB luciferase reporter gene were treated with palmitic acid, DHA or placebo, and then relative luciferase activity measured. (Panel **B**) Human SH-SY5Y neuroblastoma cells were treated with palmitic acid, DHA or placebo and then IL6 and TNFα expression was determined using adequate primers, and normalized using GAPDH. Data were presented as means ± SEM (n = 3), * and ** denoted significant differences vs control at *p* < 0.05 and *p* < 0.005, respectively.

We next investigated the effect of palmitic acid and DHA on the expression of proinflammatory cytokines IL6 and TNFα. For this purpose, SH-SY5Y cells stably expressing NFkB responsive element fused to luciferase were treated with placebo, palmitic acid or DHA. Palmitic acid significantly increased luciferase activity as compared to placebo and DHA (Fig. 2A). Furthermore, the palmitic acid but not DHA treatment significantly increased both IL6 and TNFα mRNA expression levels (Fig. 2B).

### Resistin/TLR4 signaling increased proinflammatory markers

**Figure 3:**
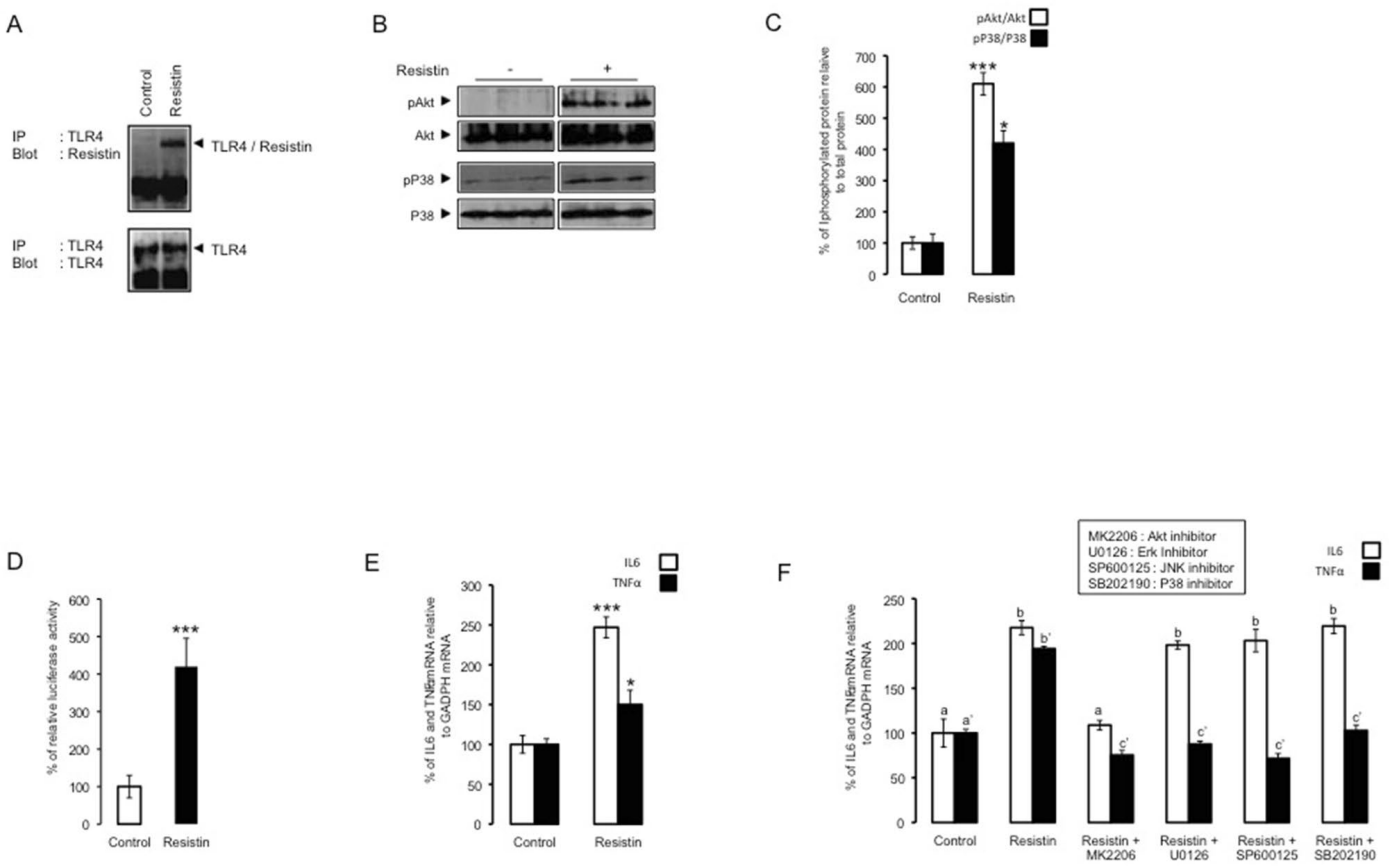
Resistin increased neuroinflammation through its binding to TLR4. (Panel **A**) Immunoprecipitation/ Immunoblot (IP/IB) analysis of the direct association of resistin with TLR4 in protein extracts from SH-SY5Y cells treated with resistin (100 ng/ml) for 16 h in the presence or absence of the cross-linker agent BS3, the shown blots are from the same membrane but blotted with different antibodies following immunoprecipitation with anti-TLR4 antibody. (Panel **B**,**C**) SH-SY5Y cells were treated with or without resistin and then Akt and p38 MAP kinase phosphorylation measured by Western blots using adequate antibodies and the presented blots was from the same membrane but blotted successively with different antibodies, the band densities were quantified and expressed as phosphorylated/total proteins. Data were presented as means ± SEM (n = 3), * and *** denoted significant differences vs control at *p* < 0.05 and *p* < 0.0005, respectively. (Panel **D**) human SH-SY5Y neuroblastoma cells stably transfected with NF-κB luciferase reporter gene were treated resistin and relative luciferase activity determined. (Panel **F**) Human SH-SY5Y neuroblastoma cells were treated with resistin in the presence or absence of Akt, Erk, JNK or p38 MAP kinase inhibitors, and then IL6 and TNFα expression was determined using specific primers for these two genes, and normalized using GAPDH. Data were presented as means ± SEM (n = 3). Columns marked by different letter differ (with or without asterix concerns TNFα or IL6, respectively) significantly (p < 0.05). For other panels, data were presented as means ± SEM (n = 3), * and *** denoted significant differences at p < 0.05 and p < 0.005, respectively. (Panel **E**) Human SH-SY5Y neuroblastoma cells were treated with resistin or placebo and then IL6 and TNFα expression was determined using adequate primers, and normalized using GAPDH. Data were presented as means ± SEM (n = 3), * and *** denoted significant differences at p < 0.05 and p < 0.001, respectively. (Panel **F**).

Based on our previous study showing that resistin acts through TLR4 in mice hypothalamus^16^, we investigated the direct interaction between resistin and TLR4 in SH-SY5Y neurons. Using cross-link approach that cross-links resistin to TLR4, which can evidence a direct binding of resistin to TLR4, we showed that resistin co-immunoprecipitates with TLR4 (Fig. 3A). In addition, resistin increased both Akt and p38 MAP kinase phosphorylation in SH-SY5Y cells (Fig. 3B,C). We also analyzed the impact of resistin on NFkB activation as well as IL6 and TNFα expression. For this purpose, using SH-SY5Y stably expressing NFkB responsive element fused to luciferase reporter gene, we show that resistin significantly increased luciferase activity (Fig. 3D). We also showed that resistin treatment increased both IL6 and TNFα expression (Fig. 3E). To decipher the specific signaling pathways involved in resistin-induced neuroinflammation, SH-SY5Y neurons were treated with resistin in the presence or absence of specific inhibitors of Akt, Erk, JNK or p38 and then IL6 and TNFα expressions. Resistin significantly increased both IL6 and TNFα expression as shown in Fig. 3E and F. The inhibition of Akt completely abolished resistin-induced up-regulation of IL6 and TNFα (Fig. 3F). In addition, resistin-induced TNFα expression was suppressed in the presence of inhibitors targeting Erk, JNK or p38 (Fig. 3F) but not that of IL6 (Fig. 3F).

### Palmitic acid but not DHA promoted resistin action

**Figure 4:**
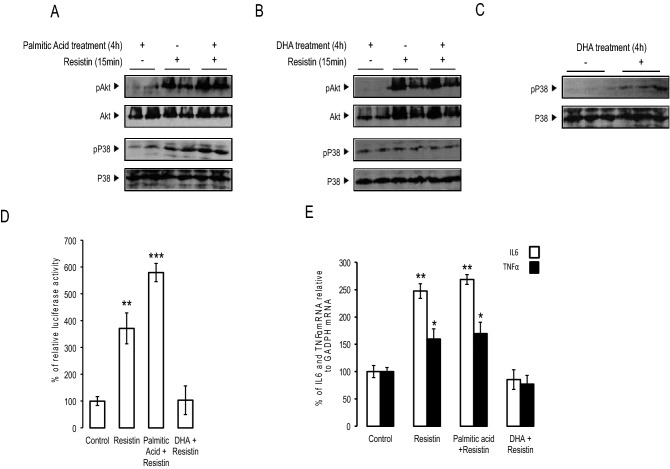
Palmitic acid promoted resistin action. Human SH-Y5Y cells were pretreated with palmitic acid (panel **A**) or DHA (panel **B**) and then stimulated by resistin. Phosphorylated Akt and p38 MAP kinase was determined by Western blot using adequate antibodies. (Panel **C**) cells were treated with DHA or placebo and p38 MAP kinase phosphorylation was determined by Western blot using adequate antibodies. The presented blots in (panel **A**), (panel **B**) and (panel **C**) are from the same membranes that were successively blotted with different antibodies. (Panel **D**) human SH-SY5Y neuroblastoma cells stably transfected with NF-κB luciferase reporter gene were treated resistin, resistin + palmitic acid or resistin + DHA, and relative luciferase activity determined. Data were presented as means ± SEM (n = 3), ** and *** denoted significant differences vs control at p < 0.005 and p < 0.001, respectively. (Panel **E**) Human SH-SY5Y neuroblastoma cells were treated with resistin, resistin + palmitic acid or resistin + DHA, and then IL6 and TNFα expression was determined using adequate primers, and normalized using GAPDH. Data were presented as means ± SEM (n = 3), * and ** denoted significant differences vs control at p < 0.05 and p < 0.005, respectively.

To study the differential effect of palmitic acid and DHA on resistin action, SH-SY5Y cells were pre-treated for 4 h with palmitic acid or DHA and then challenged with resistin. Resistin alone induced the phosphorylation of Akt and p38 MAPK (Fig. 4A). The pretreatment with palmitic acid but not DHA amplified the effect of resistin regarding Akt and p38 MAPK phosphorylation (Fig. 4A,B). We also showed that DHA pretreatment alone increased basal p38 MAPK phosphorylation (Fig. 4B,C). Then, we investigated the impact of palmitic acid or DHA pre-treatment on neuroinflammation. Thus, in SH-SY5Y cells stably expressing NFkB responsive element fused to luciferase reporter gene, we showed that palmitic acid pre-treatment amplified resistin-dependent induction of NFkB, whereas DHA abolished resistin effect (Fig. 4D). Similar results were found when IL6 or TNFα expression were measured where resistin-induced IL6 and TNFα expressions were maintained in the presence of palmitic acid and completely abolished in DHA pre-treated neurons (Fig. 4E).

### Palmitic acid but not DHA increased TLR4 location within lipid raft

**Figure 5:**
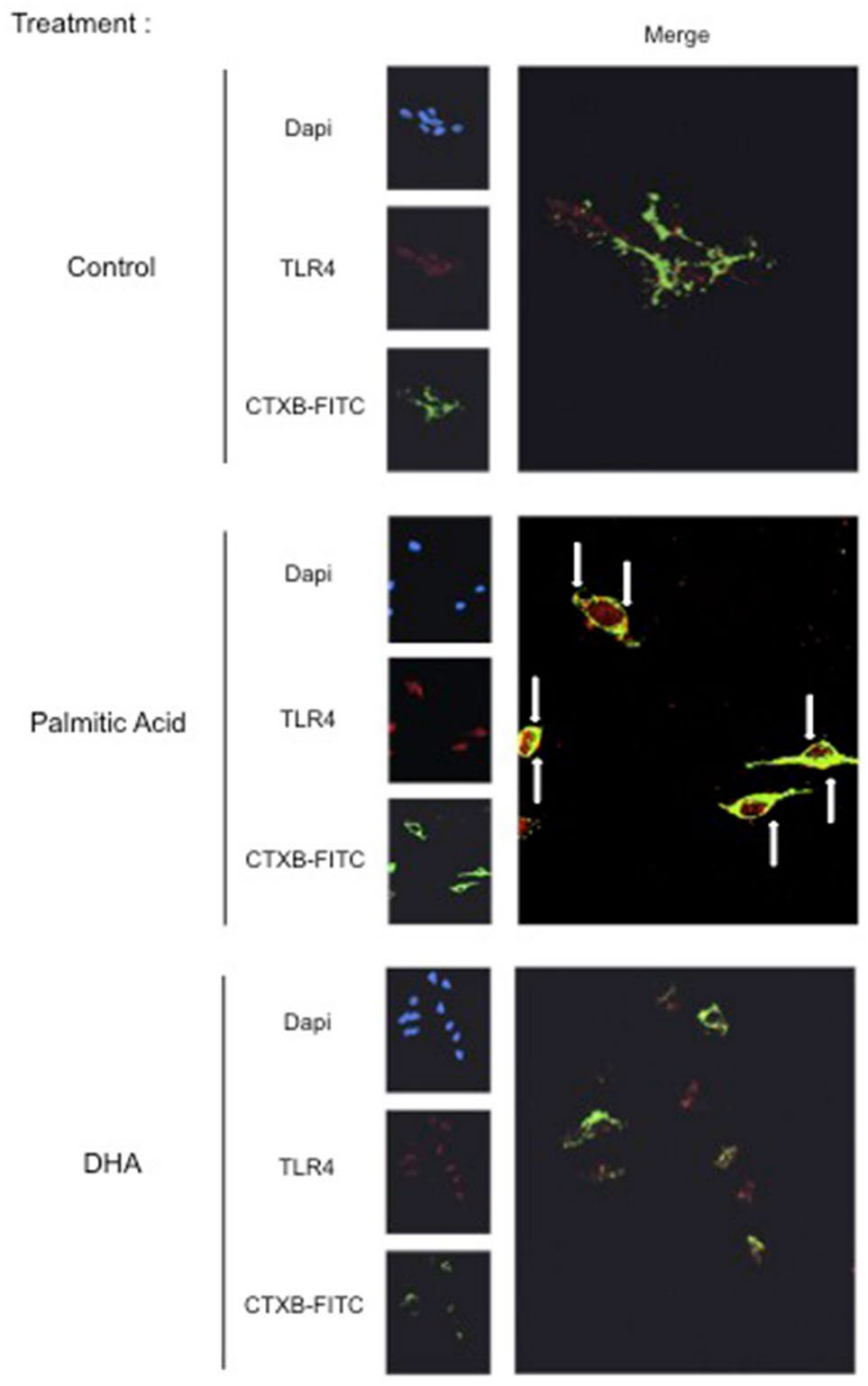
Palmitic acid induced TLR4 recruitment to membrane lipid rafts. Human SH-SY5Y cells were treated with palmitic acid, DHA or placebo during 4 h and fixed for immunohistochemistry analysis by confocal microscopy using antibodies directed towards CTXB (marker of membrane lipid rafts) or TLR4. The arrow in the merge indicated the co-localization of TLR4 and CTXB. Images were captured using a confocal microscope with high-magnification scan (X40). The figure is representative of two biological replicates and each image resulted from several slides that were analyzed by confocal microscopy.

To investigate whether palmitic acid and DHA modified TLR4 membrane location and especially within lipid rafts, we performed immunohistochemical co-localization of TLR4 and CTXB (a marker of lipid rafts). We showed that palmitic acid pretreatment increased lipid rafts and TLR4 labeling with the augmentation of TLR4 co-localization with CTXB (Fig. 5). In contrast, DHA pretreatment did not induce the co-localization of TLR4 and CTXB and did not increase TLR4 expression (Fig. 5).

### Palmitic acid required TLR4 to partially impair insulin signaling and induced inflammation

**Figure 6:**
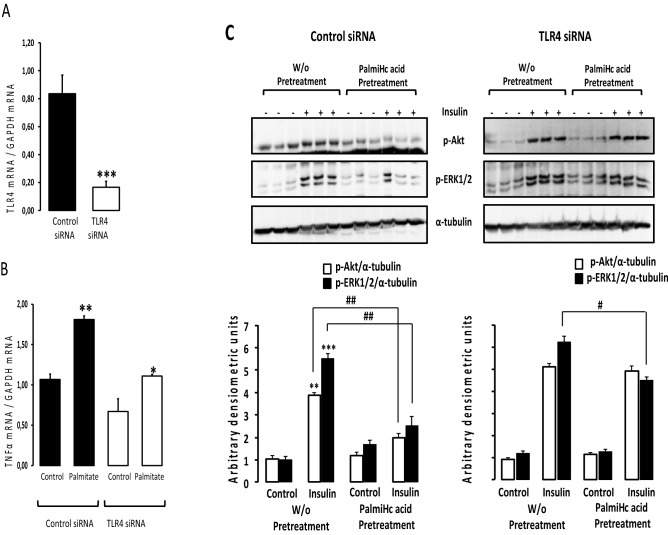
Palmitic acid required TLR4 to partially impair insulin signaling and induced inflammation. (Panel **A**) Human SH-SY5Y cells were transiently transfected with siTLR4 or scramble (control siRNA) and then TLR4 expression was measured by RT-qPCR. Data were presented as means ± SEM (n = 3). *** denoted p < 0.001 when comparing control siRNA to TLR4siRNA, statistical analysis was performed using Student t-test. (Panel **B**) Human SH-SY5Y cells were transiently transfected with siTLR4 or scramble (control siRNA), cells were cultured in the presence or absence of palmitic acid and then TNFα expression measured by RT-qPCR. Data were presented as means ± SEM (n = 3). * and ** denoted p < 0.05 and p < 0.01, respectively, when comparing control vs palmitate for both cells exposed to control or palmitate condition, statistical analysis was performed using Student t-test. (Panel **C**) Human SH-SY5Y cells transfected with control siRNA (scramble) or siTLR4 were preatreated with or without palmitic acid during 4 h then acutely stimulated by insulin. The upper part of panel C shows representative Western blots using anti-pAkt, anti-p-ERK1/2 or anti-αTubilin antibodies and each antibody was used in different membranes. The lower part of panel C shows band density quantification. Phosphorylated Akt and Erk1/2 were normalized to αTubilin. Data were presented as means ± SEM (n = 3). # and ## denoted significant differences at p < 0.05 and p < 0.01, respectively when comparing insulin-dependent phosphorylation of Akt and Erk1/2 between W/O pretreatment and palmitate treatment groups, statistical analysis was performed using Kruskal Wallis test and post-hoc Pairwise comparison in R. ** and *** denoted significant differences at p < 0.05, p < 0.1 and p < 0.001 when comparing Control vs Insulin, statistical analysis was performed using Student t-test.

In order to determine whether palmitic acid action is directly associated to neuronal TLR4 especially regarding insulin signaling and neuroinflammation, we silenced TLR4 in SH-SY5Y neurons using cells siTLR4. Firstly we showed that siTLR4 transfection significantly reduced TLR4 expression in SH-SY5Y cells (Fig. 6A). The down-regulation of TLR4 significantly attenuated palmitic acid-dependent augmentation of TNFα expression (Fig. 6B). Furthermore, palmitic acid pretreatment significantly reduced insulin-dependent Akt and Erk1/2 phosphorylation (Fig. 6C) and this effect was partially abolished in cells expressing siTLR4 (Fig. 6C).

### Palmitic acid increased resistin-dependent TLR4/MYD88/TIRAP complex formation

**Figure 7:**
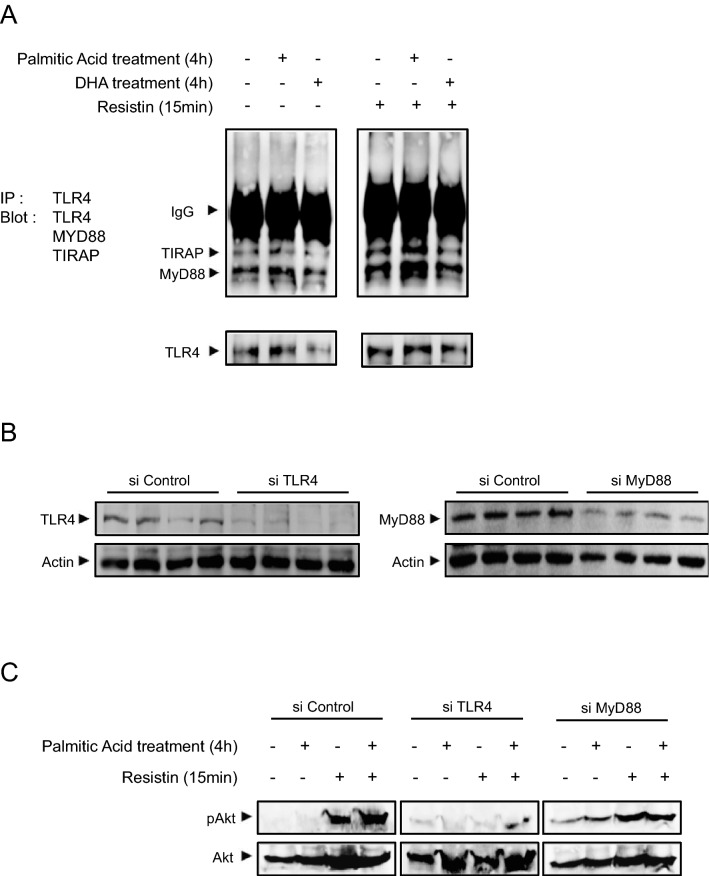
Palmitic acid increased resistin-dependent TLR4/MYD88/TIRAP association. (Panel **A**) Immunoprecipitation/Immunoblot (IP/IB) showing the association of TLR4 with MyD88 and TIRAP was analyzed in human SH-SY5Y neuroblastoma cells following treatment with palmitic acid or DHA and in response to resistin by Western blot using adequate antibodies. All blots are from the same membrane. Each experiment has been performed in triplicates as technical replicates. (Panel **B**) Human SH-SY5Y cells were transfected with scramble (Control siRNA), siTLR4 or siMyD88 and then TLR4 and MyD88 expression were measured by Western blot normalized to actin, for each antibody different blots were used. Each experiment has been performed in triplicates as technical replicates. (Panel **C**) Palmitic acid, resistin or placebo either treated these transfected cells and then Western blots were performed using anti-pAkt or Akt. These blots were performed in independent membranes. Each experiment has been performed in triplicates as technical replicates.

In order to determine whether palmitic acid promoted the activation of resitin/TLR4 signaling, we investigated the impact of palmitic acid and DHA on the formation of the signaling complex TLR4/MYD88/TIRAP in response to resistin. Resistin alone increased the co-immunoprecipitation of TLR4/TIRAP and TLR4/MYD88 (Fig. 7A). Following palmitic acid pretreatment, resistin effect was amplified as evidenced by the augmentation of TLR4/TIRAP and TLR4/MYD88 co-immunoprecipitation (Fig. 7A). DHA pre-treatment did not increase resistin-dependent TLR4/TIRAP/MYD88 co-immunoprecipitation but rather decreased it especially concerning TIRAP (Fig. 7A). To further investigate resistin signaling through TLR4/MYD88, SH-SY5Y cells lacking TLR4 or MYD88 were generated by siRNA methodology. Firstly, we verified that siTLR4 and siMYD88 cells exhibited a strong reduction of TLR4 and MYD88 expression, respectively (Fig. 7B). In siTLR4 cells, resistin was unable to activate Akt phosphorylation even following palmitic acid pre-treatment (Fig. 7C). Similar result was obtained in siMYD88 cells but however with a lesser extent as compared to that obtained in siTLR4 cells (Fig. 7C).

## Discussion

Plasma FFA levels are elevated in most Type2 Diabetes and obese subjects contributing to the loss of insulin responsiveness especially at the peripheral level^18,19^. Indeed, in pancreatic β cells saturated fatty acids such palmitic acid inhibited insulin gene expression and pancreatic β cells’ functions^11^. In endothelial cells, palmitic acid reduced insulin-dependent Akt phosphorylation and increased ER stress through the down-regulation of endoplasmic reticulum calcium ATPase (SERCA)^20^. Furthermore, FFAs have been also associated to increased inflammation in various cellular models and *in vivo*^21–25^. The inflammatory effects of FFAs have been linked to the activation of TLR4 in pancreatic β cells^11^, immune cells and adipocytes^13^. More recently, palmitic acid has been described as an inducer of resistin gene expression and secretion in 3T3-L1 adipocytes^13^. These studies suggest that the FFAs exert their deleterious effects through TLR4 but so far the direct binding of FFAs to TLR4 has not been demonstrated. Furthermore, TLR4 has been reported to be the binding site for LPS leading to inflammatory responses^14,26,27^. We reported that resistin, an adipokine promoting insulin resistance, initiates its effects through the binding with TLR4 at the neuronal level leading to pro-inflammatory response and insulin resistance^15^, raising the question whether and to what extent resistin action depends on palmitic acid or other FFAs. So far only limited number of studies have described such connection. Indeed, resistin acutely impaired insulin-stimulated glucose transport and Akt phosphorylation, in isolated rat soleus muscles only in the presence of palmitic acid^28^. However, at either peripheral or neuronal level, the potential impact of palmitic acid on resistin/TLR4 signaling remains undocumented. Here, we deciphered the mechanisms linking palmitic acid to resistin/TLR4 signaling pathway on SH-SY5Y neuronal cells. Firstly we confirmed our previous finding that showed the interaction of resistin with TLR4 in mouse hypothalamus^15^; indeed we showed that resistin binds to TLR4 on SH-SY5Y cells. We also showed that palmitic acid impaired insulin-dependent Akt phosphorylation whereas DHA maintained insulin action. Our results are in good agreement with numerous studies reporting the pro-inflammatory action of palmitic acid and saturated FFA at the peripheral^11,20,21,29^ or neuronal level^30,31^. Here, we showed that the effect of palmitic acid is concomitant with its action on TLR4 expression, indeed palmitic acid up-regulated TLR4 expression in SH-SY5Y neurons and in contrast DHA seemed to not affect its expression. Indeed, it has been reported that palmitic acid promoted inflammation through TLR4 accessory protein MD2 in myocardial cells and human dendritic cells^32,33^. However, the up-regulation of TLR4 in response to palmitic acid has not been previously described. According to these data we could suggest that by inducing the expression of neuronal TLR4, palmitic acid promoted resistin action. As a matter of fact, we showed that resistin-induced neuroinflammation was amplified in the presence of palmitic acid, and interestingly resistin effects were abolished in the presence of DHA. We have also shown that resistin-induced neuroinflammation is dependent upon Akt signaling pathway. It is noteworthy to indicate that when applied acutely both insulin and resistin induced Akt phosphorylation through insulin signaling pathways and resistin/TLR4 pathway, respectively, as we have previously reported^15^. However, when neurons were chronically exposed to resistin, insulin-induced Akt phosphorylation is abolished. Indeed, when cells are stimulated with resistin in an acute manner (10 to 15 min), resistin is able to induce Akt phosphorylation as a part of TLR4 signaling pathway. However, when cells are exposed to resistin for long term (2 h to 16 h), resistin is able to induce the expression of negative regulators of insulin signaling such as SOCS-3, PTP-1B and JNK as we have previously reported^15^. In addition, we may suggest, especially concerning resistin acute effects, that resistin could signal through a specific Akt isoform since 3 Akt isoforms are described and expressed by different genes. This needs further investigations to differentiate between insulin and resistin signaling regarding the recruited Akt isoform. Furthermore, Akt converges both metabolic and inflammatory pathways as reported in macrophage^34^ and added to the fact that there are three Akt isoforms (Akt1, Akt2 and Akt3) we may suggest that resistin and insulin could signal through different Akts and this is still undocumented. Interestingly, we identified the potential mechanism by which palmitic acid could reinforced and amplified resistin action through most likely the recruitment of TLR4 to plasma membrane rafts and the augmentation of the resistin dependent TLR4/MYD88/TIRAP complex formation. In contrast, DHA did not affect TLR4 recruitment to lipid rafts or resistin signaling pathway.

In conclusion, we report, for the first time to our knowledge, that resistin-induced insulin resistance and neuroinflammation are potentiated by neuronal exposure to palmitic acid and strongly attenuated by DHA treatment. The potential cross-talk between palmitic acid or FFAs in general and resistin could constitute a potential mechanism involved in the onset of neuronal insulin resistance and inflammation, and could reflect the close relationship between inappropriate diet consumption and altered adipose tissue adipokines’ secretions such that of resistin. Thus, it is of interest to perform further investigations in vivo.

## Supplementary Information

Supplementary Information
