# Publisher Correction: Palmitic acid promotes resistin‑induced insulin resistance and inflammation in SH‑SY5Y human neuroblastoma

**DOI:** 10.1038/s41598-021-92151-w

**Published:** 2021-06-15

**Authors:** Hamza Amine, Yacir Benomar, Mohammed Taouis

**Affiliations:** 1 Molecular Neuroendocrinology of Food Intake (NMPA), CNRS UMR 9197, University of Paris-Saclay, Orsay, France; 2 NMPA, Department of Development, Evolution and Cell Signaling, CNRS UMR 9197, Paris-Saclay Institute of Neurosciences (NeuroPSI), Orsay, France; 3 Functional Genomics and Cancer, IGBMC, Illkirch, France

Correction to: *Scientific Reports* 10.1038/s41598-021-85018-7, published online 08 March 2021

The original version of this Article contained errors.

In Figure 1B,C and Figure 3C,D,E, the x-axis and y-axis values in the bar graphs were omitted.

Additionally, in Figure 5, the white arrow indicators for the co-localization of TLR4 and CTXB in the image for Palmitic Acid were incorrectly captured.

The original Figures 1, 3 and 5 and accompanying legends appear below.

**Figure 1:**
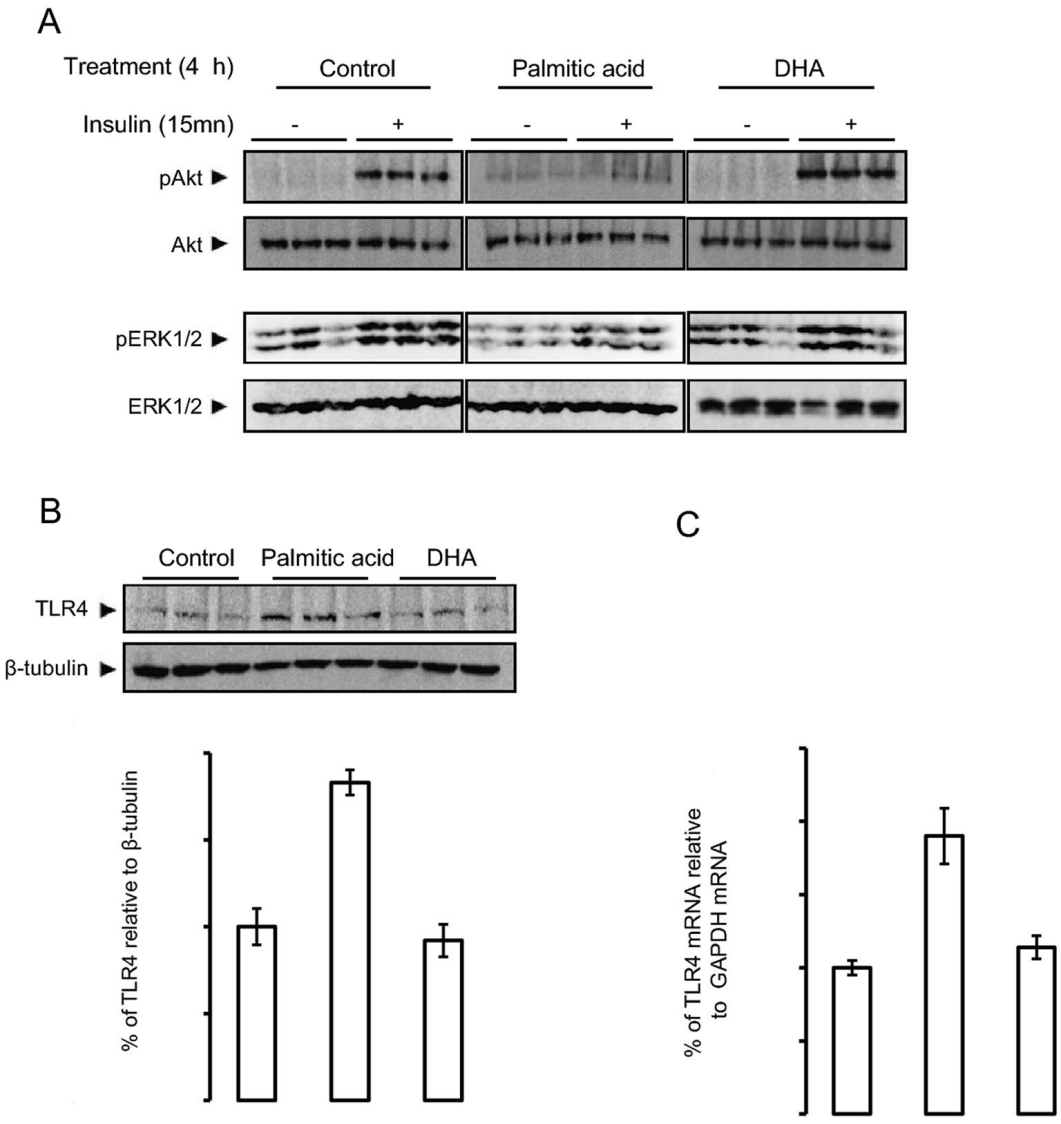
Palmitic acid but not DHA, inhibits insulin action and up-regulates TLR4. Human SH-Y5Y neuroblastoma cells were pretreated with palmitic acid, DHA or placebo during 4 h, then insulin-dependent Akt and ERK1/2 phosphorylation measured by Western blots using adequate antibodies, Control, Palmitic acid and DHA blots were performed in separate membranes, in each case the same membrane was blotted with different antobodies (panel **A**); TLR4 expression was measured by Western blot using anti-TLR4 antibodies and the expression was normalized to β tubilin, and band densities were quantified and expressed as ratio of TLR4/β-tubilin (panel **B**); TLR4 mRNA expression was determined by qRT-PCR normalized to GAPDH (panel **C**). Western blot and qRT-PCR data were presented as means ± SEM (n = 3), * and ** denoted significant differences vs control at *p* < 0.05 and *p* < 0.005, respectively.

**Figure 3:**
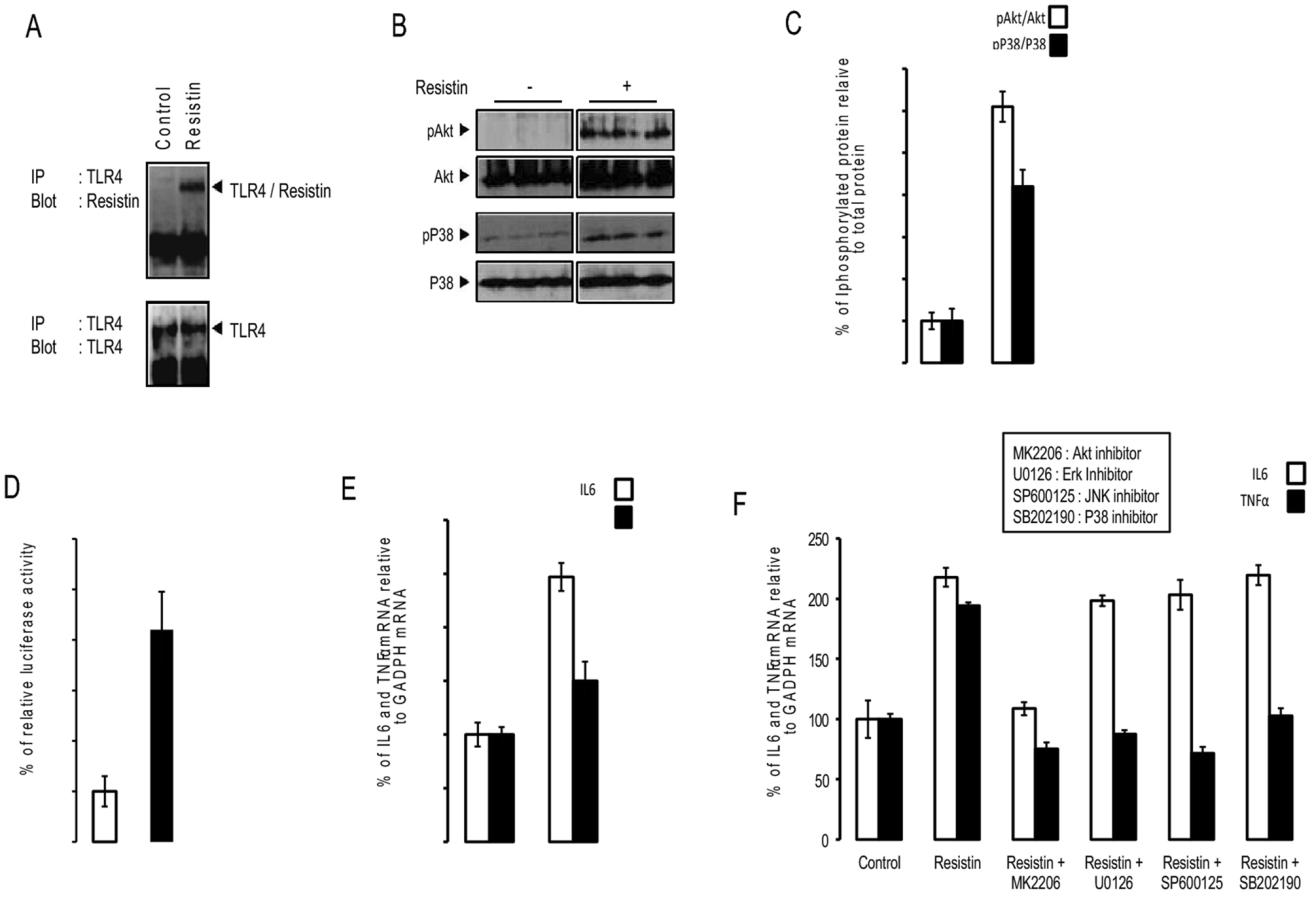
Resistin increased neuroinflammation through its binding to TLR4. (Panel **A**) Immunoprecipitation/Immunoblot (IP/IB) analysis of the direct association of resistin with TLR4 in protein extracts from SH-SY5Y cells treated with resistin (100 ng/ml) for 16 h in the presence or absence of the cross-linker agent BS3, the shown blots are from the same membrane but blotted with different antibodies following immunoprecipitation with anti-TLR4 antibody. (Panel **B**,**C**) SH-SY5Y cells were treated with or without resistin and then Akt and p38 MAP kinase phosphorylation measured by Western blots using adequate antibodies and the presented blots was from the same membrane but blotted successively with different antibodies, the band densities were quantified and expressed as phosphorylated/total proteins. Data were presented as means ± SEM (n = 3), * and *** denoted significant differences vs control at *p* < 0.05 and *p* < 0.0005, respectively. (Panel **D**) human SH-SY5Y neuroblastoma cells stably transfected with NF-κB luciferase reporter gene were treated resistin and relative luciferase activity determined. (Panel **F**) Human SH-SY5Y neuroblastoma cells were treated with resistin in the presence or absence of Akt, Erk, JNK or p38 MAP kinase inhibitors, and then IL6 and TNFα expression was determined using specific primers for these two genes, and normalized using GAPDH. Data were presented as means ± SEM (n = 3). Columns marked by different letter differ (with or without asterix concerns TNFα or IL6, respectively) significantly (p < 0.05). For other panels, data were presented as means ± SEM (n = 3), * and *** denoted significant differences at p < 0.05 and p < 0.005, respectively. (Panel **E**) Human SH-SY5Y neuroblastoma cells were treated with resistin or placebo and then IL6 and TNFα expression was determined using adequate primers, and normalized using GAPDH. Data were presented as means ± SEM (n = 3), * and *** denoted significant differences at p < 0.05 and p < 0.001, respectively. (Panel **F**).

**Figure 5:**
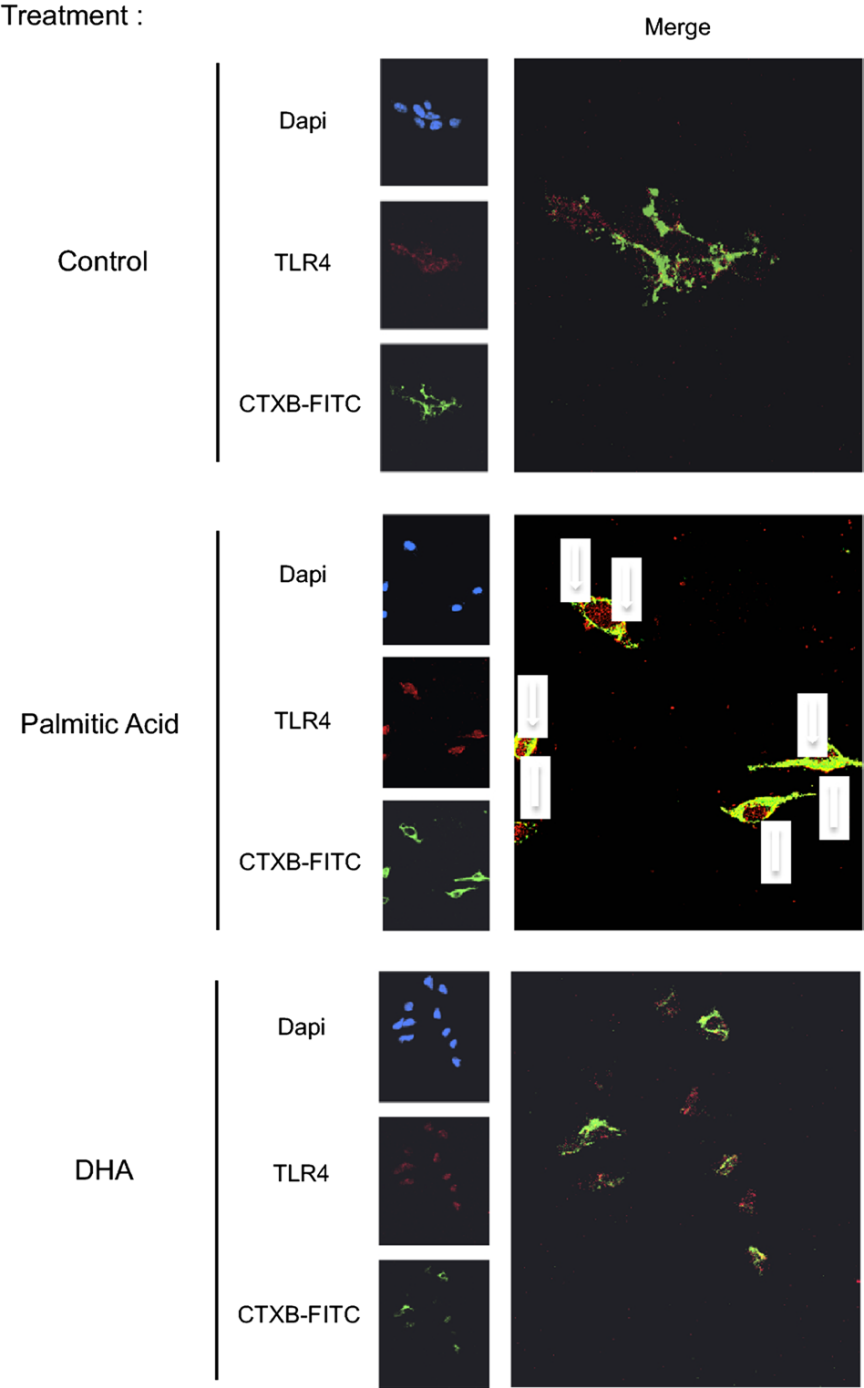
Palmitic acid induced TLR4 recruitment to membrane lipid rafts. Human SH-SY5Y cells were treated with palmitic acid, DHA or placebo during 4 h and fixed for immunohistochemistry analysis by confocal microscopy using antibodies directed towards CTXB (marker of membrane lipid rafts) or TLR4. The arrow in the merge indicated the co-localization of TLR4 and CTXB. Images were captured using a confocal microscope with high-magnification scan (X40). The figure is representative of two biological replicates and each image resulted from several slides that were analyzed by confocal microscopy.

The original Article has been corrected.

