# Supplementary material for: Palmitic acid promotes resistin-induced insulin resistance and inflammation in SH-SY5Y human neuroblastoma
Source: Sci Rep. 2021 Mar 8;11:5427. doi: 10.1038/s41598-021-85018-7 (PMC7940652; doi:10.1038/s41598-021-85018-7)
Supplement: Supplementary file 1 — Supplementary Information [file 41598_2021_85018_MOESM1_ESM.pptx]

## Slide 1
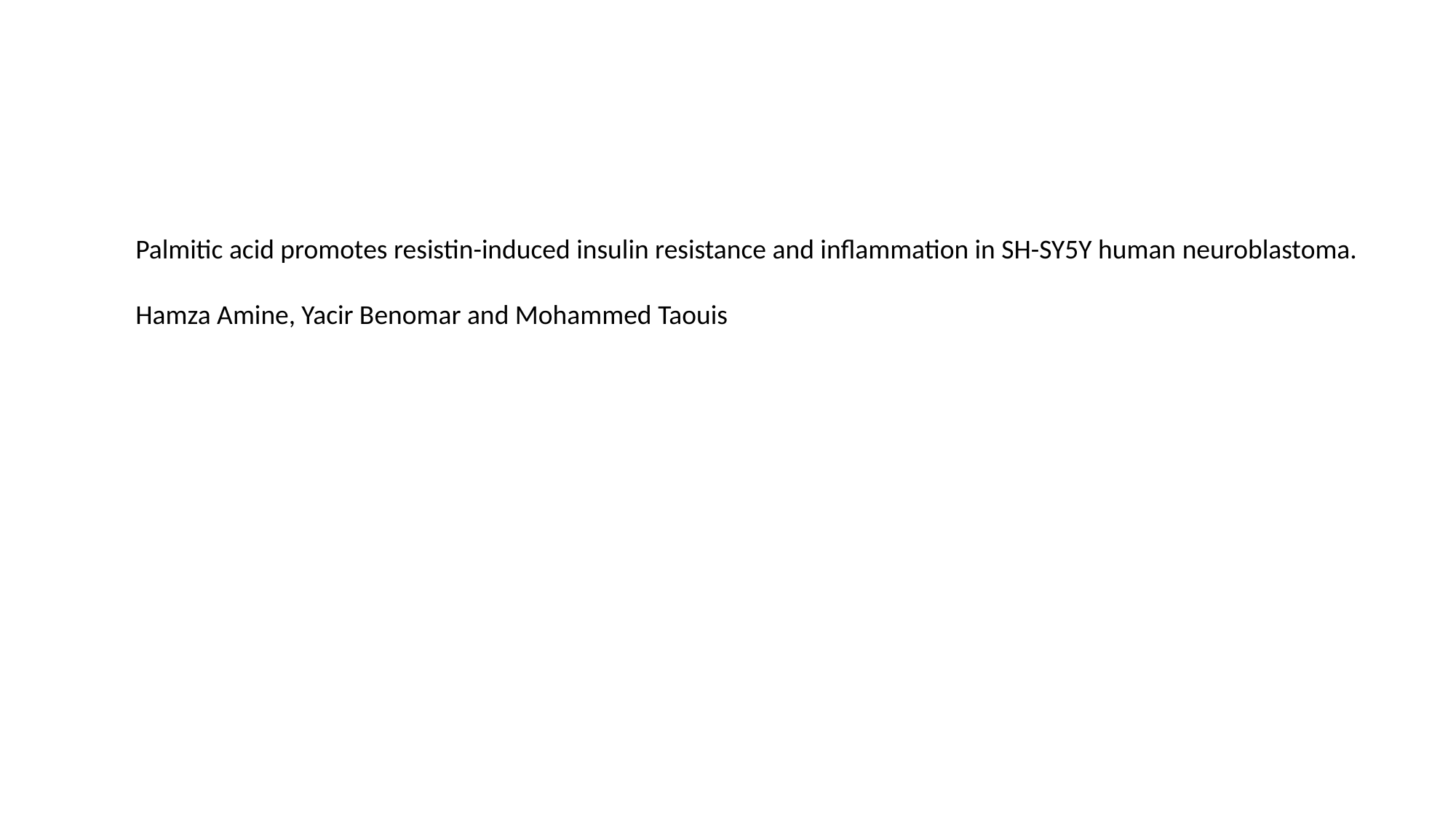

Palmitic acid promotes resistin-induced insulin resistance and inflammation in SH-SY5Y human neuroblastoma.
Hamza Amine, Yacir Benomar and Mohammed Taouis

## Slide 2
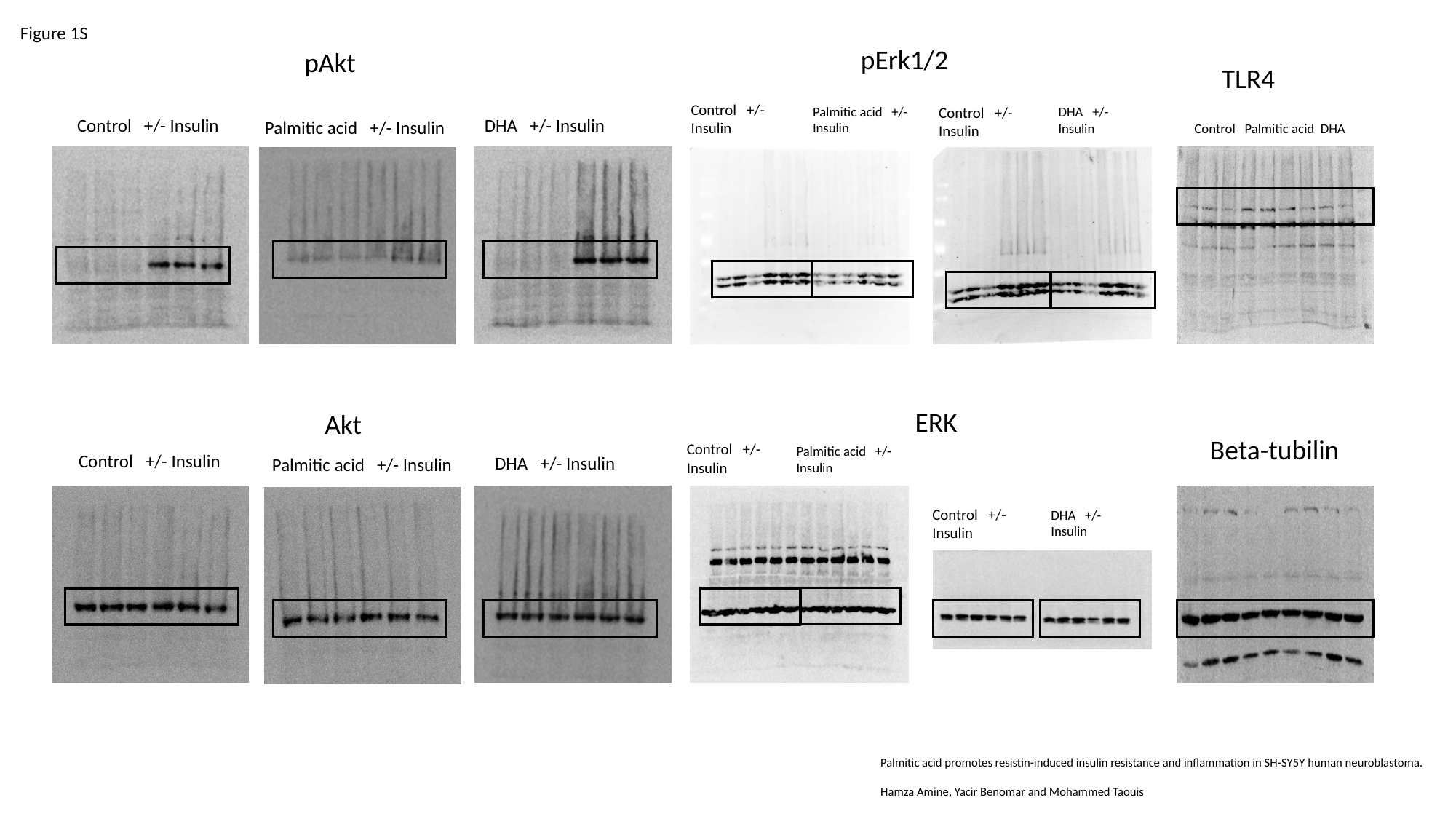

Figure 1S
pErk1/2
pAkt
TLR4
Control +/- Insulin
Palmitic acid +/- Insulin
Control +/- Insulin
DHA +/- Insulin
Control +/- Insulin
DHA +/- Insulin
Palmitic acid +/- Insulin
Control Palmitic acid DHA
ERK
Akt
Beta-tubilin
Control +/- Insulin
Palmitic acid +/- Insulin
Control +/- Insulin
DHA +/- Insulin
Palmitic acid +/- Insulin
Control +/- Insulin
DHA +/- Insulin
Palmitic acid promotes resistin-induced insulin resistance and inflammation in SH-SY5Y human neuroblastoma.
Hamza Amine, Yacir Benomar and Mohammed Taouis

## Slide 3
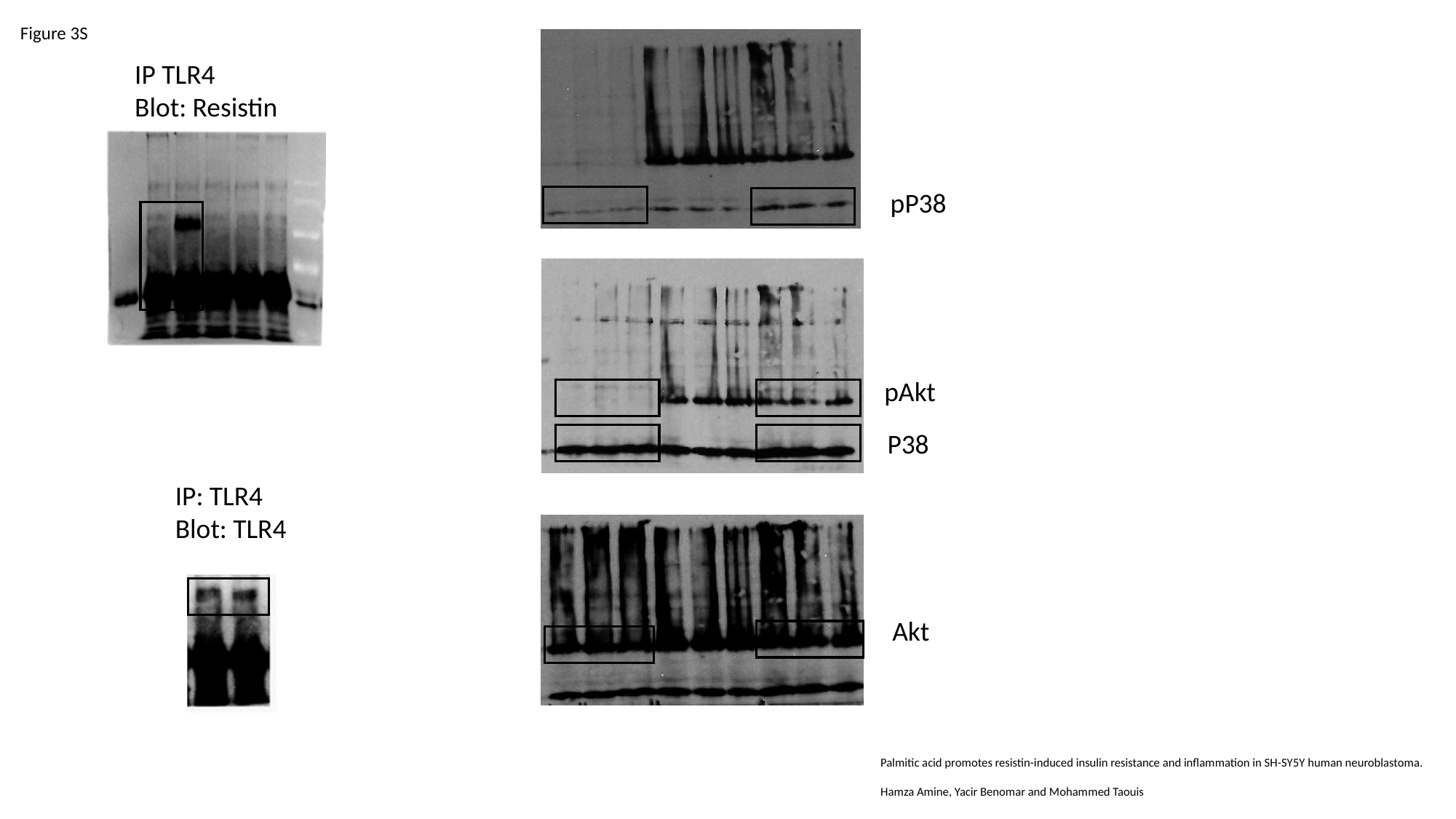

Figure 3S
IP TLR4
Blot: Resistin
pP38
pAkt
P38
IP: TLR4
Blot: TLR4
Akt
Palmitic acid promotes resistin-induced insulin resistance and inflammation in SH-SY5Y human neuroblastoma.
Hamza Amine, Yacir Benomar and Mohammed Taouis

## Slide 4
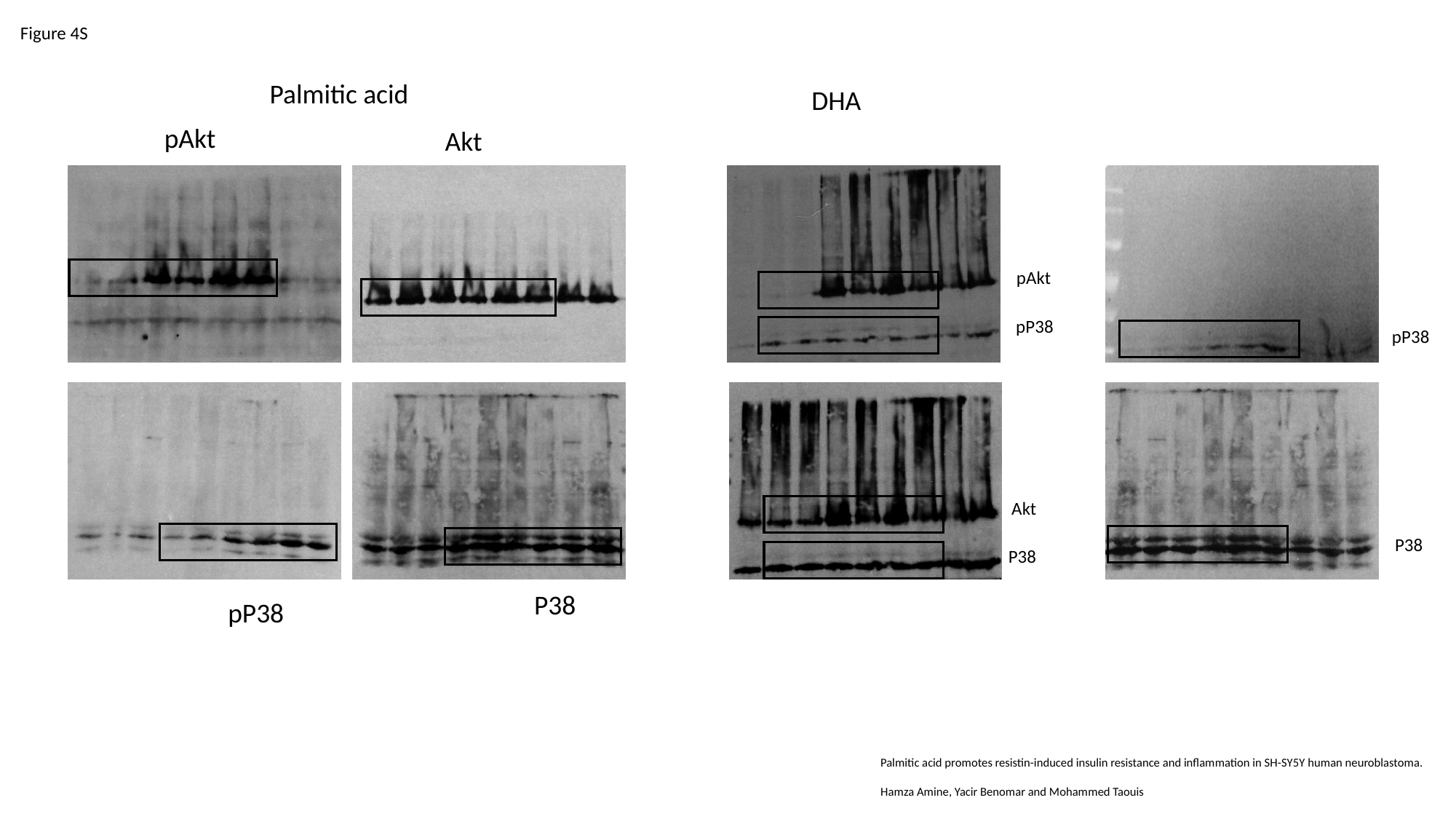

Figure 4S
Palmitic acid
DHA
pAkt
Akt
pAkt
pP38
pP38
Akt
P38
P38
P38
pP38
Palmitic acid promotes resistin-induced insulin resistance and inflammation in SH-SY5Y human neuroblastoma.
Hamza Amine, Yacir Benomar and Mohammed Taouis

## Slide 5
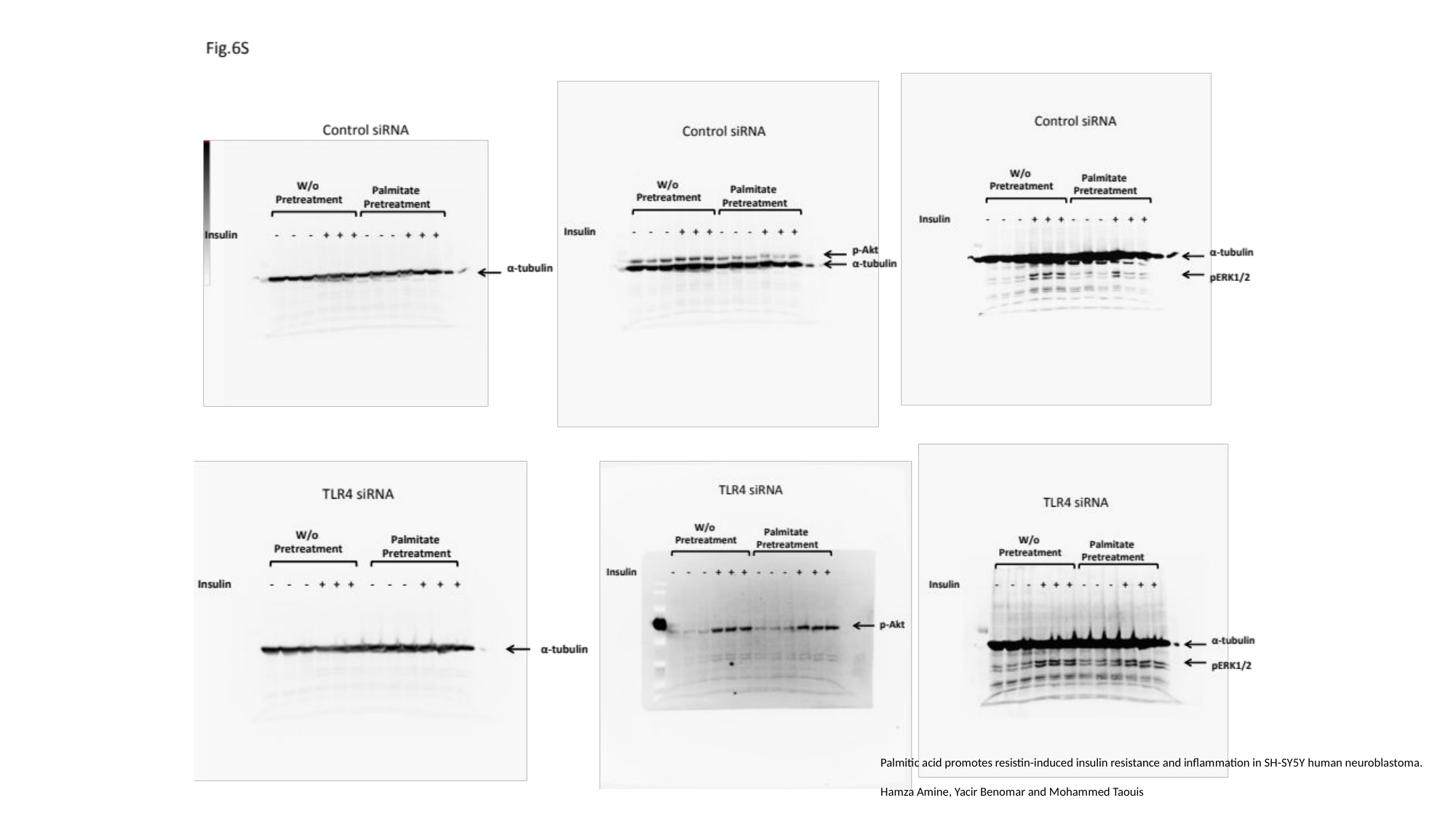

Palmitic acid promotes resistin-induced insulin resistance and inflammation in SH-SY5Y human neuroblastoma.
Hamza Amine, Yacir Benomar and Mohammed Taouis

## Slide 6
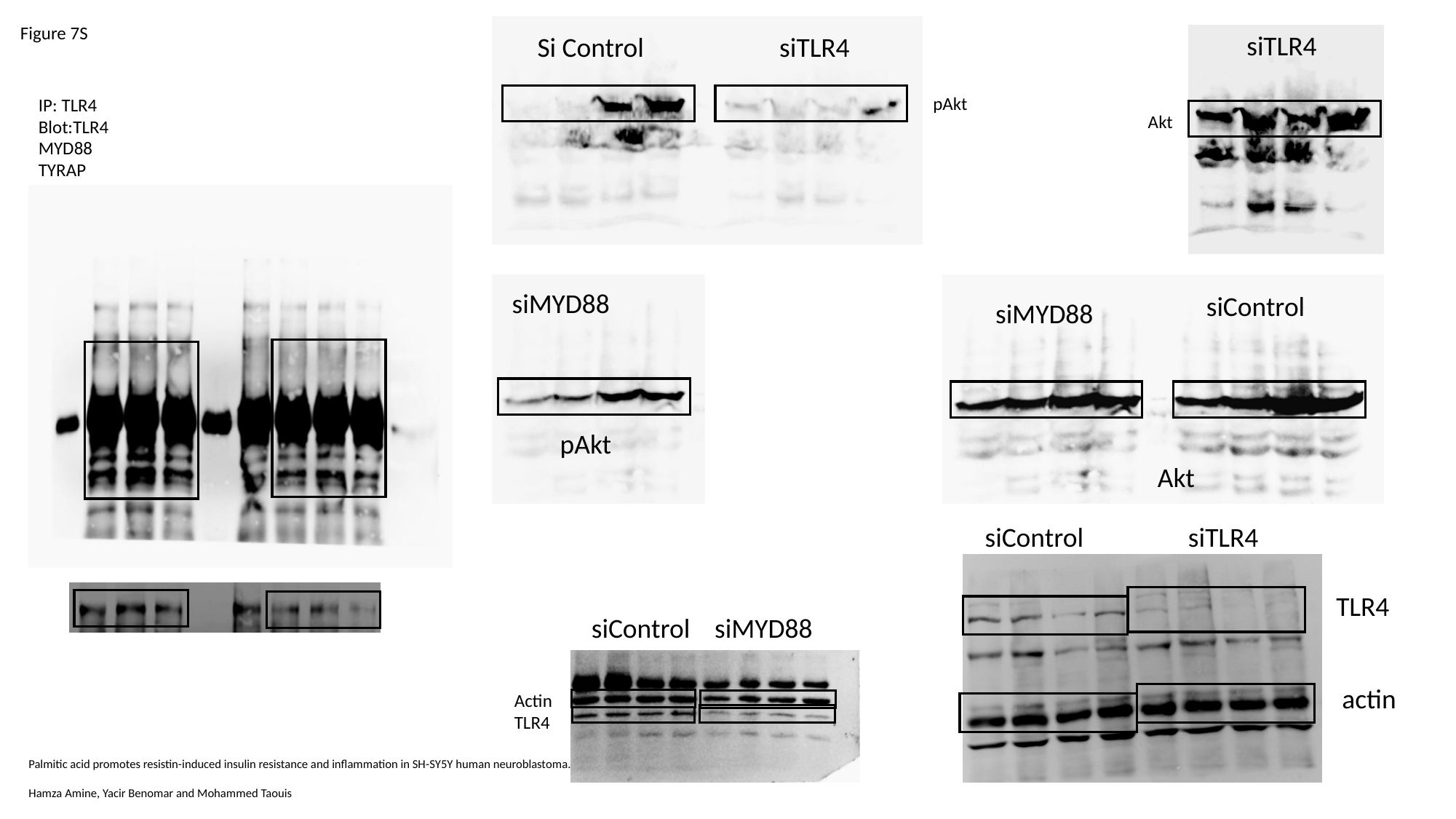

Figure 7S
siTLR4
Si Control siTLR4
pAkt
IP: TLR4
Blot:TLR4
MYD88
TYRAP
Akt
siMYD88
siControl
siMYD88
pAkt
Akt
 siControl siTLR4
TLR4
siControl siMYD88
actin
Actin
TLR4
Palmitic acid promotes resistin-induced insulin resistance and inflammation in SH-SY5Y human neuroblastoma.
Hamza Amine, Yacir Benomar and Mohammed Taouis

## Slide 7
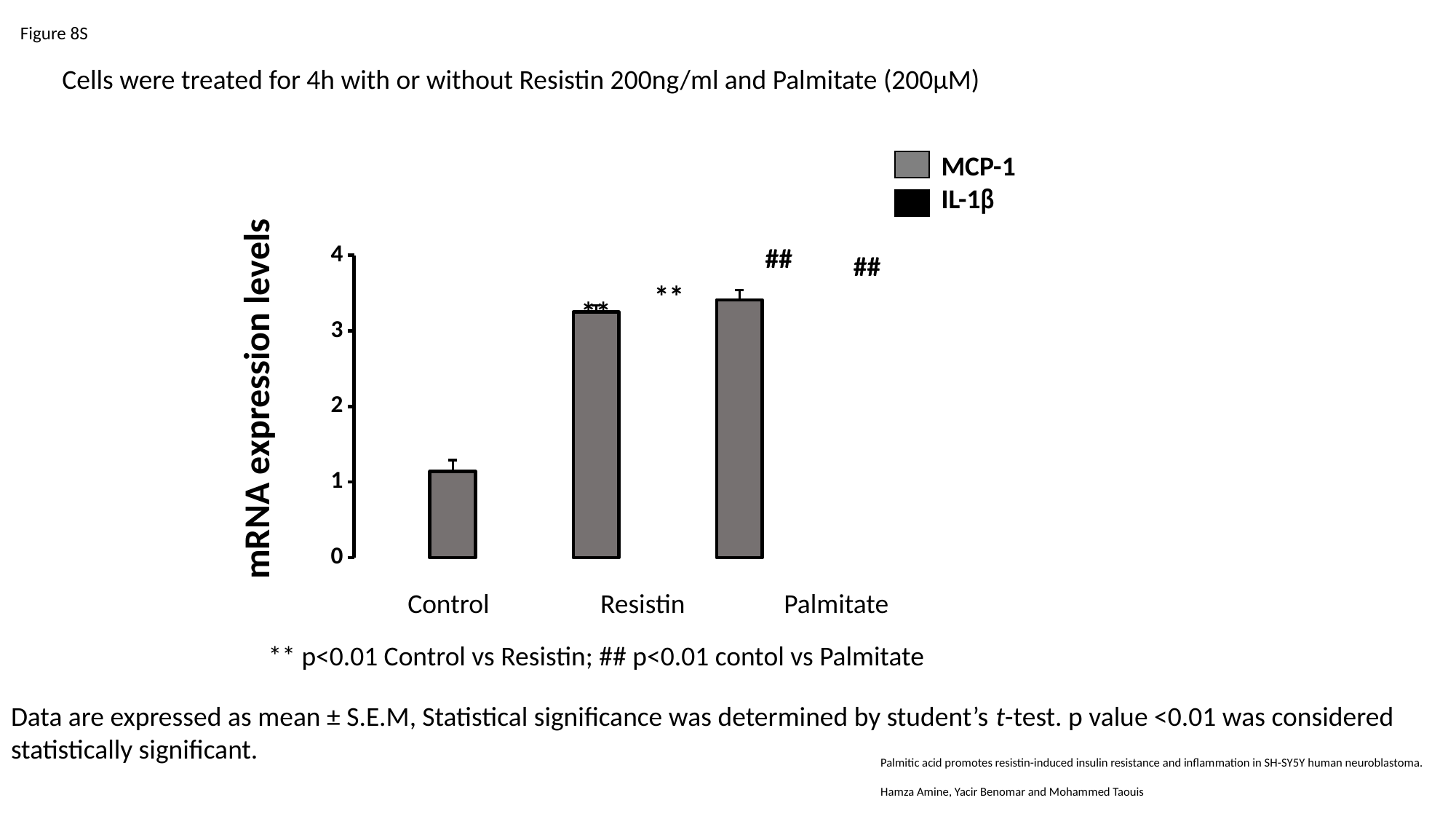

Figure 8S
Cells were treated for 4h with or without Resistin 200ng/ml and Palmitate (200µM)
MCP-1
IL-1β
##
### Chart
| Category | il1b | mcp1 |
|---|---|---|##
 **
**
mRNA expression levels
Control Resistin Palmitate
** p<0.01 Control vs Resistin; ## p<0.01 contol vs Palmitate
Data are expressed as mean ± S.E.M, Statistical significance was determined by student’s t-test. p value <0.01 was considered statistically significant.
Palmitic acid promotes resistin-induced insulin resistance and inflammation in SH-SY5Y human neuroblastoma.
Hamza Amine, Yacir Benomar and Mohammed Taouis
